# *Lecanicillium psalliotae* (Hypocreales: Cordycipitaceae) Exerts Ovicidal and Larvicidal Effects against the Sheep Blood-Feeding Nematode *Haemonchus contortus* through Its Liquid Culture Filtrates

**DOI:** 10.3390/pathogens13070588

**Published:** 2024-07-16

**Authors:** Gustavo Pérez-Anzúrez, Pedro Mendoza-de Gives, Miguel Ángel Alonso-Díaz, Elke von Son-de Fernex, Adolfo Paz-Silva, María Eugenia López-Arellano, Agustín Olmedo-Juárez

**Affiliations:** 1 Laboratory of Helminthology, National Centre for Disciplinary Research in Animal Health and Innocuity (CENID-SAI), National Institute for Research in Forestry, Agriculture and Livestock, INIFAP-SADER, Jiutepec 62550, Mexico; tavopzaz@gmail.com (G.P.-A.); mlopez_arellano@hotmail.com (M.E.L.-A.); aolmedoj@gmail.com (A.O.-J.); 2 Production Sciences and Animal Health, Faculty of Veterinary Medicine and Zootechnics, National Autonomous University of Mexico, Coyoacán 04510, Mexico; 3 Tropical Livestock Center, Faculty of Veterinary Medicine and Zootechnics, National Autonomous University of Mexico, Martínez de la Torre 93600, Mexico; alonsodma@hotmail.com (M.Á.A.-D.); elkevsf@hotmail.com (E.v.S.-d.F.); 4 Department of Animal Pathology, Faculty of Veterinary, University of Santiago de Compostela, 27142 Lugo, Spain; adolfo.paz@usc.es

**Keywords:** *Lecanicillium*, nematophagous fungi, alkaloids, tannins, ovicidal, larvicidal

## Abstract

Nematophagous fungi (NF) form part of the soil microbiota and are natural enemies of nematodes, helping to regulate nematode populations. A verticillate NF isolated from soil from Tepalcingo, Mexico, was morphologically and molecularly characterised. This fungus was cultured in two different liquid media—Czapek-Dox broth (CzDoxB) and sweet potato dextrose broth (SPDB)—for 21 days. The ovicidal (OA) and larvicidal (LA) activities of fungal liquid culture filtrates (LCFs) were assessed in 96-well microtitre plates at different concentrations against *Haemonchus contortus* after 48 h. The morphological and molecular identification revealed the presence of *Lecanicillium psalliotae*. Additionally, the groups of compounds associated with nematocidal activity were determined from a qualitative chemical profile (QCP) using different reagents. The highest OA of the LCFs was obtained at 25 mg/mL from SPDB and CzDoxB and amounted to 97.2 and 99.06%, respectively. Meanwhile, the highest LA recorded with these LCFs at 100 mg/mL was 54.27% and 96.8%, respectively. The QCP revealed the presence of alkaloids and tannins in both LCFs that have previously been associated with nematocidal activity. *Lecanicillium psalliotae* exerted an important effect on *H*. *contortus* and could be of significance in future studies focused on the control and prevention of haemonchosis in small ruminants.

## 1. Introduction

Soil mycobiota possess an extraordinary variety of micro-fungi that play an important ecological role in the recycling of nitrogen and carbon [1,2]. Micro-fungi carry out many ecosystem services, together with other microbial communities (mainly Bacteria and Archaea), and they are vital to maintaining equilibrium in soil ecosystems [3]. Micro-fungi have several ecological responsibilities, one of which is regulating the populations of other organisms [4]. Micro-fungi act as biological regulators of insects [5,6], bacteria [7], other fungi [8], protozoa [9], and some nematodes, i.e., free-living nematodes and plant and animal parasitic nematodes [10,11]. 

The fungus *Lecanicillium psalliotae* is a verticillated soil micro-fungus belonging to the phylum Ascomycota, the class Sordariomycetes, the order Hypocreales, and the family Cordycipitaceae [12]. *Lecanicillium psalliotae* inhabits different ecological sites, such as woodlands and agricultural soils [10]. This species has been found to be an entomopathogenic fungus infecting insects and feeding on their internal tissues [13]. It can also be found as a nematophagous fungus living in the proximity of galled roots of legumes and vegetables infected with root-knot nematodes. The fungus penetrates the females and eggs of species of the phytoparasite *Meloidogyne*, e.g., *M*. *mayaguensis*, and eventually feeds on the nematode embryo [14]. A recent study reported the high ovicidal and larvicidal activity of *L. psalliotae* against *M. javanica* under in vitro and in vivo conditions in tomato crops [15]. Nematodes are another type of soil microorganism that play an important role in the food chain, degrading nitrogen and carbon in soil. They comprise a group of worms living in different ecosystems, from the warm tropics to cool Antarctica, and they have adapted to different lifestyles. Some species live in soil or in aqueous substrates as free-living nematodes [16]. Others have adapted to life as parasitic organisms, infecting animals [17], plants [18,19], or humans [20].

The nematode *Haemonchus contortus* is a blood-feeding parasite that lives its adult stage in the abomasum (stomach) of small grazing ruminants [21]. This parasite can cause severe anaemia that can lead to the loss of appetite, weight loss, emaciation, the diminishment of the immune system, susceptibility to diseases, and even the death of young animals [22,23]. Conventional treatments against sheep and goat haemonchosis are based on the frequent and continuous administration of chemical anthelmintic drugs; however, this method has undesirable consequences, such as leaving chemical residues in milk, meat, or other parts meant for human consumption, making this a public health problem [24]. Likewise, the drugs administered into the animals are eliminated through urine and faeces, which threatens beneficial soil microorganisms, becoming an environmental risk [25,26]. Additionally, all commercially available chemical anthelmintic drugs trigger the imminent development of anthelmintic resistance in parasites [27,28]. The objective of this study was to assess the in vitro ovicidal and larvicidal activity of the fungus *L*. *psalliotae* liquid culture filtrates against the sheep blood-feeding nematode *H*. *contortus*.

## 2. Materials and Methods

### 2.1. Location

This study was performed at the Laboratory of Helminthology of the National Centre for Disciplinary Research in Animal Health and Innocuity (CENID-SAI) belonging to the National Institute for Research in Agriculture, Forestry and Livestock, Agriculture, Mexican Government, Jiutepec Municipality, Morelos State, Mexico.

### 2.2. Haemonchus contortus Eggs and Infective Larvae

The eggs and infective larvae of the parasitic nematode were obtained using faeces taken from the rectum of a lamb artificially infected with the parasite and maintained in the Laboratory of Helminthology at CENID-SAI. The pre-patent period was monitored to collect and analyse the faeces of this animal, searching for the presence of parasite eggs using the flotation technique. Once the presence of eggs was identified, the faeces were homogenised with water and passed through two meshes with 74 and 37 μm pore diameters. The eggs were collected from the last mesh and washed several times with water. Subsequently, the eggs were cleaned using differential gradient centrifugation with 40% sucrose solution for 5 min and washed three times using centrifugation for 3 min with distilled water to eliminate the sucrose residues.

Faeces containing parasite eggs were processed using the coproculture technique and maintained for seven days to allow for egg hatching and larval growth until L_3_. Infective larvae were recovered from coprocultures using the Baermann funnel method. Once larvae were obtained, they were cleaned following the same procedure used to clean the eggs of the parasite, the differential gradient technique via centrifugation, and then the larvae were unsheathed using 0.187% sodium hypochlorite solution for 3–5 min and washed to remove the sheaths [29]. Clean eggs were used to perform the in vitro egg-hatching inhibition assay [30], and clean larvae were employed for the larval mortality assay [31].

### 2.3. Fungal Isolation

Fungus isolation was performed using soil and root samples taken from a fig (*Ficus carica*) crop infested with root-knot nematodes (*Meloidogyne* spp.) located in the Municipality of Tepalcingo, Morelos state, Mexico (latitude 18°34′45″ N; longitude 98°49′00″ W). The samples were processed in the Laboratory of Helminthology at CENID-SAI in Jiutepec Municipality, Morelos State, Mexico. The soil and roots were placed on the surface of water agar sterile plates, and the plates were incubated at laboratory temperature (18–30 °C). The plates were viewed daily under the microscope searching for characteristic structures of verticillated fungi (Sordariomycetes), i.e., phialides, verticillated conidiophores, macro- and microconidia, and the presence or absence of chlamydospores. Once these structures were identified in one of our plates, fungal material was transferred to sterile rose bengal agar plates. Taxonomic structures were again transferred to new sterile plates. This process was repeated until plates with the pure fungus were obtained.

### 2.4. Fungal Morphological Identification

The isolated fungus was cultured following the microculture method: a square (1 cm^2^) of potato dextrose agar (PDA) was taken and placed on a slide that was supported on a glass Petri dish with 10 mL of distilled water. The fungus was inoculated on each side of the agar square, and a coverslip was placed on the surface. Microcultures were inoculated for some days until the fungus grew up to cover the coverslip [32]. The coverslips were collected, placed on a slide with a water drop or cotton blue staining, and observed under a microscope to measure characteristic structures, i.e., macro- and microconidia, verticillated conidiophores, and phialides, using a LEICA DM6-B microscope (LEICA, Wetzlar, Germany). A total of 50 measurements of each structure were recorded (conidia length and width, phialide length and width, and number of phialides/conidiophore). The obtained data were compared with those for species reported by other authors to determine the isolated species [33].

### 2.5. Fungal Molecular Identification

The fungus was cultured on potato dextrose broth (PDB) (Merck KGaA, Darmstadt, Germany) using 250 mL flasks and incubated for 15 days at room temperature (18–25 °C). The DNA was obtained from fungal mycelia using the Wizard^®^ Genomic DNA Purification Kit (Promega, Madison, WI, USA). The quantification of genetic material was achieved using an IMPLEN spectrophotometer (NanoPhotometer NP80, Munich, Germany). Additionally, the ITS-4 (5′-GGAAGTAAAAGTCGTAACAAGG-3′) and ITS-5 (5′-TCCTCCGCTTATTGATATGC-3′) oligonucleotides were selected to amplify the DNA ITS region [34], and the EF-1018F (GAYTTCATCAAGAACATGAT) and EF-1620R (GACGTTGAADCCRACRTTGTC) primers were used to amplify the TEF1-α region [35] in PCR. A C1000 Touch Thermal Cycler (Bio-Rad, Hercules, CA, USA) was used.

The PCR technique was carried out according to the following conditions: for ITS, initial denaturation at 94 °C for 3 min, 35 cycles of denaturation at 94 °C for 1 min, annealing at 42 °C for 90 s, extension at 72 °C for 90 s, and a final extension stage at 72 °C for 5 min; for TEF1-α, there was initial denaturation at 95 °C for 5 min, 35 cycles of denaturation at 95 °C for 1 min, annealing at 51 °C for 60 s, extension at 72 °C for 120 s, and a final extension stage at 72 °C for 10 min. The genomic product sizes were confirmed using electrophoresis on 1.5% agarose gel, and the Wizard^®^ SV Gel and PCR Clean-Up System (Promega, Madison, WI, USA) was used to purify the obtained amplicons following the manufacturer’s recommendations. The resulting genic material was sent to the Institute of Biotechnology of the National University Autonomous of Mexico (IBT-UNAM) to be sequenced using an Applied Biosystem Sequencer (Thermo Fisher Scientific, Waltham, MA, USA).

Once the sequences were obtained, they were aligned using the Basic Alignment Search Tool (BLAST, https://blast.ncbi.nlm.nih.gov/Blast.cgi, accessed on 17 April 2024). Sequences from *Lecanicillium* and related species for ITS and TEF1-α were obtained from the NCBI database, and multiple alignments for each region were developed using MEGA software (v.11.0.13). A total of 31 sequences were employed for the analysis. The best substitution models were estimated using JModelTest software (v.2.1.10). Both regions were linked using MEGA and BioEdit software (v.5.0.9). Maximum likelihood and Bayesian analysis were performed in IQTree (v.1.6.12) and MrBayes (v.3.2.6) software, respectively. Bootstrap values (BT) were obtained by ultrafast bootstrapping, and posterior probabilities (PP) were obtained by MCMC analysis, with a total of 1,000,000 replicates. The best tree was visualised in FigTree (v.1.3.1) and edited using Nitro Pro (v.13.9.1).

### 2.6. Fungal Culture in Liquid Media

The fungus *L*. *psalliotae* was cultured in two liquid media: sweet potato dextrose broth (SPDB) and Czapek-Dox broth (CzDoxB). SPDB was prepared as follows: A quantity of 200 g of organic sweet potato was cut in squares of 1 cm (approximately) and boiled in a precipitate glass with 1 L of distilled water. The solid material was separated by sieving using gauze. The liquid was recovered into a precipitated glass (one litter volume) and 20 g of dextrose was added and mixed. Finally, the liquid was adjusted to a one-litre volume with distilled water. CzDoxB was prepared by dissolving 35 g of the medium (Sigma-Merck, Darmstadt, Germany, in powder) in 1 L of distilled water. Fifty millilitres of the medium (SPDB or CzDoxB) were transferred to 250 mL volume flasks (three flasks per medium). The entire set of flasks was sterilised using an autoclave for 20 min at 121 °C. Once the media were cool, three cylindrical plugs (1 cm diameter) from a 2-week-old *L*. *psalliotae* isolate grown on PDA were transferred to each flask and incubated at room temperature (18–25 °C) under static conditions for 21 days.

### 2.7. Fungal Liquid Culture Filtrates

The liquid culture filtrates (LCFs) were obtained by filtration using coffee filter paper in a funnel. This step was performed to separate the mycelia from the liquid material. The liquid fraction was filtered through a Whatman paper # 4 (25 µm pore diameter) (Merck, KGaA, Darmstadt, Germany). The resulting liquid was passed through a 2 µm glass fibre filter (Millipore, KGaA, Darmstadt, Germany). Finally, two filtration processes were performed using 0.45 and 0.22 µm nitrocellulose filters (Millipore, KGaA, Darmstadt, Germany). This last step guaranteed that our final LCF was sterile and only contained the myco-compounds produced by our isolate. LCFs were concentrated and dried before the assay. This step was achieved using a rotatory evaporator (Büchi R-300, Flawil, Switzerland) and a lyophiliser (Labconco, Kansas, MO, USA). After lyophilisation, the dried filtrates were reconstituted with water or phosphate-buffered saline (PBS) to assess the ovicidal and larvicidal activity, respectively. After the procedure, the LCFs were ready to be assessed.

### 2.8. Assessment of the In Vitro Nematocidal Activity of Lecanicillium psalliotae against Haemonchus contortus

The ovicidal assessment was performed using 96-well microtitre plates. Fifty microliters of the corresponding LCF and 50 μL of an aqueous suspension containing 100 *H*. *contortus* eggs (approximately) were added to each well (n = 4). The LCFs of *L*. *psalliotae* produced in two media (SPDB and CzDoxB) and at four concentrations (10, 15, 20, and 25 mg/mL) were assessed. Likewise, two series of wells (n = 4) containing only distilled water and the media (without any fungi) were considered negative controls. The results for the confrontation eggs/LCF were obtained after 48 h of exposure.

The results were based on counting the total number of hatched and non-hatched eggs under a light microscope at a 5× objective. The egg hatch inhibition percentage was estimated after comparing the hatched and non-hatched eggs in the treated and control groups. The entire experiment was repeated in triplicate.

#### Assessing the Larvicidal Activity of *Lecanicillium psalliotae* Liquid Culture Filtrates against *Haemonchus contortus*

The larvicidal assessment was also performed using 96-well microtitre plates. Fifty microliters of the corresponding LCF and 50 μL of PBS (pH = 7.2) suspension containing 100 *H*. *contortus* larvae (approximately) were added to each well (n = 4). The liquid culture filtrates of *L*. *psalliotae* produced in the two media (SPDB and CzDoxB) and at three concentrations (25, 50, and 100 mg/mL) were assessed. Likewise, two series of wells containing only PBS (pH = 7.2) and the media (without any fungi) were considered negative controls. The results for the confrontation larvae/LCF were obtained after 48 h of exposure.

The results were based on counting the total number of motionless and moving larvae under a light microscope at 5× and 10× objectives. The motionless larvae were touched with a metallic fine needle as a stimulus to promote their movement, if still alive. If larvae remained motionless after being touched with the needle, they were considered dead [36]. The larval mortality percentage was estimated after comparing the means of live and dead larvae recovered from both experimental groups.

### 2.9. Microphotographic Record

A set of microphotographs was taken to show possible morphological changes in eggs and larvae attributed to the effect of exposure to LCFs using a LEICA DM6-B microscope (LEICA, Wetzla, Germany).

### 2.10. Myco-Chemical Profile

The profile of myco-compounds was identified using standard phytochemical test techniques with the proper reagents and methods. To identify the presence of alkaloids, Dragendorff, Mayer, and Wagner’s reagents were used. Likewise, the Bornträger test was selected to identify the presence of coumarins. Flavonoids were identified using Mg^2+^ and HCl tests. The presence of tannins was identified using ferric chloride, gelatine, and saline solution tests. Additionally, the Lieberman–Burchard and Salkowski tests were used to determine the presence of triterpenes and sterols. Finally, the presence of saponins was determined from the occurrence of foam formation. The myco-chemical profile was conducted according to Delgado-Núñez et al., 2020 [37].

### 2.11. Statistical Analysis

Data from egg-hatching and larval mortality assays were analysed using an analysis of variance (ANOVA) (*p* < 0.05), and mean multiple comparison was performed using Tukey’s test. Different concentrations were compared in the same medium (*p* < 0.05). Additionally, logistic regression using the PROBIT method was performed to determine the lethal concentrations 50 (LC_50_) and 90 (LC_90_).

## 3. Results

### 3.1. Fungal Morphological Taxonomy

**Figure 1:**
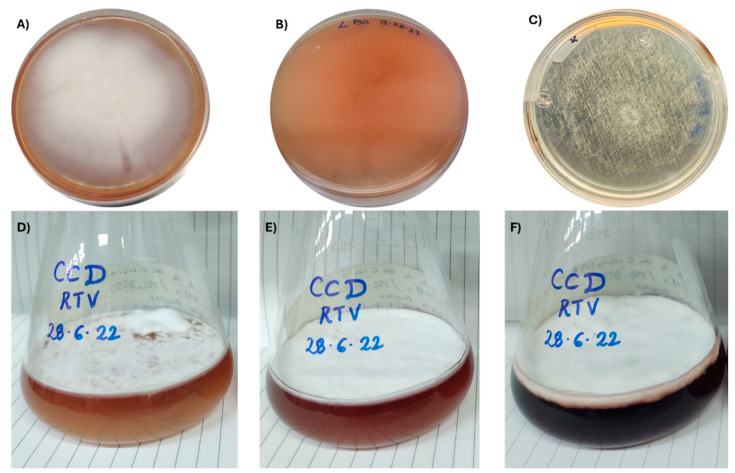
Aspect of the *Lecanicillium psalliotae* INIFAP-STp-01 strain growing in potato dextrose agar plates; (**A**) front and (**B**) reverse, and (**C**) the fungus growing in water agar (front view); (**D**–**F**) the fungus growing in a flask containing sweet potato dextrose broth at 2, 3, and 15 days.

Macroscopically, after 21 days at room temperature (18–25 °C), plates with the fungus growing on PDA showed a white, cottony colony on the front, with a light reddish colour on the opposite side (Figure 1A,B). In water agar, slow and poor growth was observed (Figure 1C). This strain grew much faster in liquid cultures, covering the whole surface (8 cm in diameter) after only 3 days following inoculation at room temperature (18–25 °C). Interestingly, this fungus grew mostly on the surface of both media, rather than being submerged. It is interesting that the medium was initially yellow but turned light red on the day after inoculation, and the medium colour gradually changed to deep red over approximately 7 days (Figure 1D–F).

**Figure 2:**
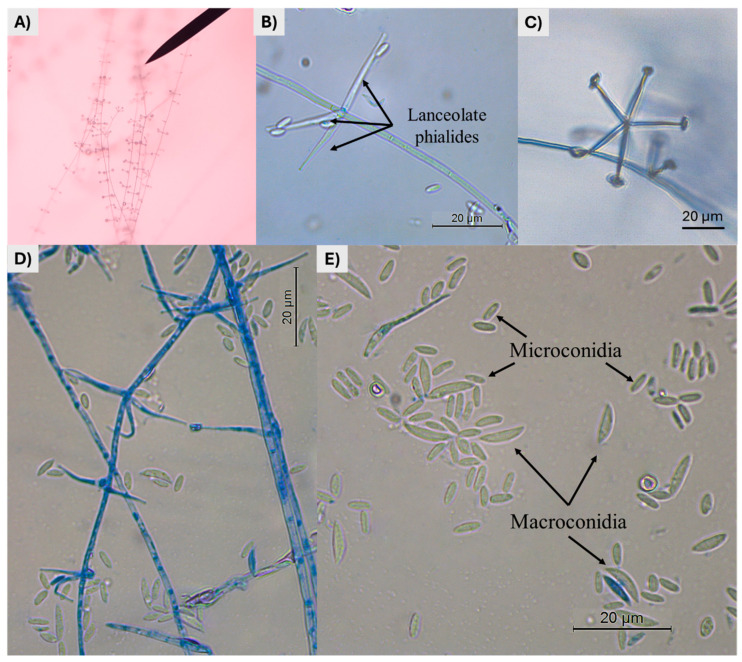
Microphotographs from *Lecanicillium psalliotae* showing: (**A**) Aerial prostrate hyphae, with several phialides forming whorls; (**B**,**C**) a whorl with three and five lanceolate phialides, crowned for a cluster of macro- and microconidia; (**D**) coloured hyphae with verticillated conidiophores with phialides (cotton blue staining was used); (**E**) macroconidia falcate with pointed ends, slightly curved, and microconidia, oval to ellipsoidal, with rounded ends.

Microscopically, our isolate showed the following morphological characteristics: a net of prostrate aerial hyphal ties (Figure 2A); several verticillated conidiophores with lanceolate phialides, solitary or in whorls of two to five, each phialide crowned with a macroconidium, and solitary or in clusters with microconidia (Figure 2B–D); macroconidia falcate to ellipsoidal, slightly curved (6.74–11.94 µm × 1.56–2.61 µm); and microconidia oval to ellipsoidal, straight or slightly curved (4.27–5.73 µm × 1.25–2.04 µm) (Figure 2E; Table 1).

### 3.2. Molecular Taxonomy

**Figure 3:**
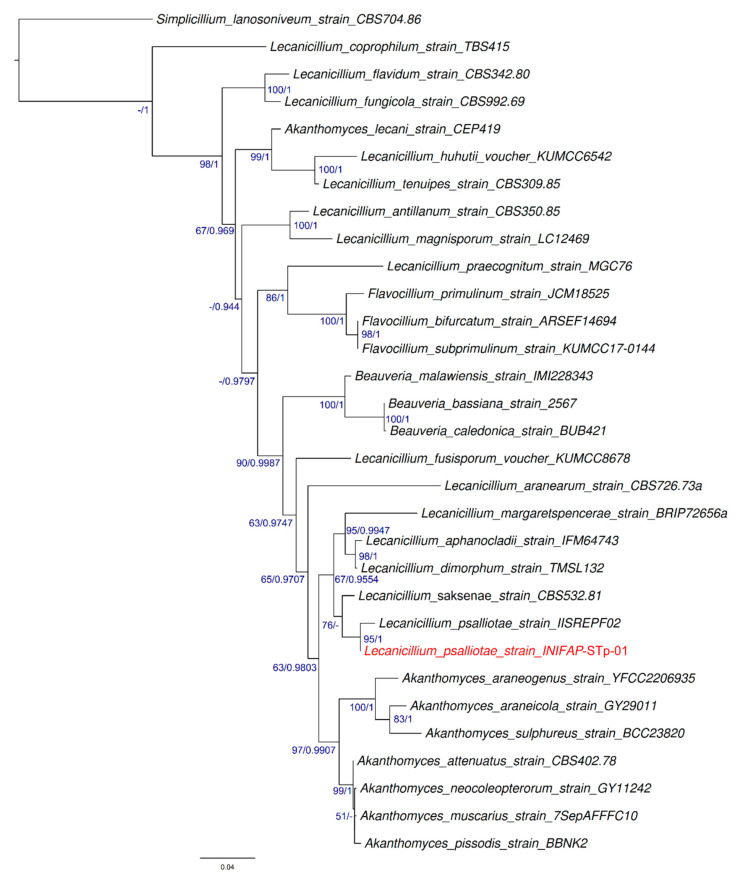
Phylogenetic analysis from the *Lecanicillium psalliotae* INIFAP-STp-01 strain (in red) and related species using 31 linked sequences from the ITS and TEF1-α regions. Support values >50% and >0.9 for Maximum Likelihood and Bayesian Inference, respectively, are shown in nodes (in blue).

The alignment of our sequences using the BLASTn tool showed high coverage (99%) and similarities between our isolate and species from the *Lecanicillium* genus: *L*. *saksenae* (99.66%) and *L*. *psalliotae* (99.31%). However, when the TEF1-α region was aligned, the best score was obtained with *L*. *psalliotae* (KF358375) (99.82% similarity). The constructed phylogenetic tree showed that our strain had a common predecessor with the *L*. *psalliotae* species (KF358375). High values of support were obtained for this node (BB = 95 and PP = 1.00), and both were related to *L*. *saksenae* (EF469067) (Figure 3).

### 3.3. Ovicidal Activity of Fungal Liquid Culture Filtrates against Haemonchus contortus

The means and standard errors of *H*. *contortus* eggs and pre-infective larvae recovered after exposure to different LCF concentrations from *L*. *psalliotae* cultured in SPDB and CzDoxB, as well as the egg-hatching inhibition percentages, are shown in Table 2. It should be noted that the process of obtaining *H*. *contortus* eggs involves taking the fresh faeces of an artificially infected egg-donor sheep containing eggs of the parasite and must be conducted quickly, since under the conditions established in our laboratory (18–25 °C), the eggs form an embryo and hatch the first larval stage (L_1_) in approximately 24 h. In the present experiment, in the controls (LCF-free), almost the entire sample of eggs succeeded in hatching larvae during this period.

Likewise, the PROBIT analysis by logistic regression showed that the LC_50_ was 9.856 and 10.625 mg/mL for SPDB and CzDoxB, respectively. Meanwhile, the LC_90_ was 17.797 and 19.454 mg/mL, respectively.

**Figure 4:**
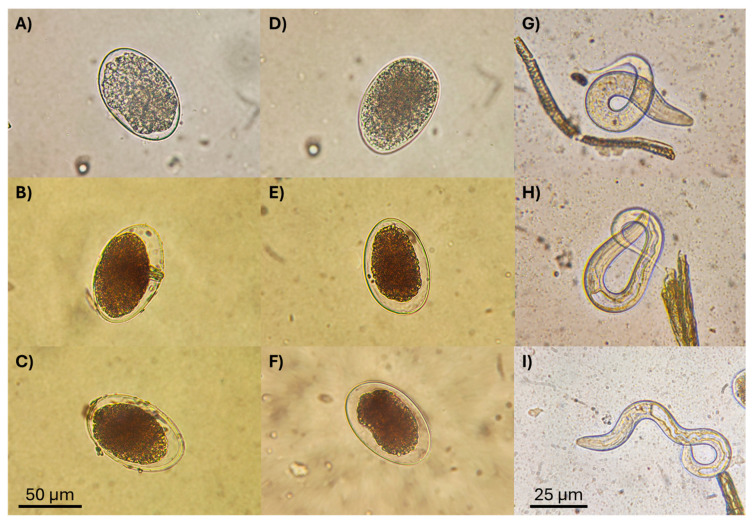
Microphotographs showing *Haemonchus contortus* eggs with null embryo development after 48 h of being exposed to *Lecanicillum psalliotae* liquid culture filtrates cultured in sweet potato dextrose broth (**B**,**C**) and in Czapek Dox Broth (**E**,**F**), eggs at the beginning of the experiment (**A**,**D**), and larvae hatched from control groups (**G**–**I**). Bar scale of 50 µm is for eggs, and that of 25 µm is for larvae.

After the exposure of *H*. *contortus* eggs to the *L*. *psalliotae* LCF, most eggs showed an undeveloped state, with most not hatching in either LCF (Figure 4B,C,E,F). In contrast, most of the non-exposed eggs (control) hatched, and many first larval stages were seen vigorously moving into the well (Figure 4G–I). The microscopical analysis of the *H*. *contortus* eggs exposed to LCF showed no apparent changes on the egg-coat surface, which appeared smooth; however, most of the eggs had stopped their development at an early stage, at which time neither a well-defined morula nor embryo were observed. Additionally, the egg cell mass appeared destroyed and showed a diffuse aspect, and an abnormal space between the cellular mass of the egg and the internal egg coat was observed. Most of the few larvae that successfully developed and reached their hatching process appeared dead or remained alive only for a few hours.

### 3.4. Nematocidal Activity of Fungal Liquid Culture Filtrates against Haemonchus contortus Infective Larvae

The results of the nematocidal activity of the three concentrations of LCF from *L*. *psalliotae* in both SPDB and CzDoxB against *H*. *contortus* infective larvae are shown in Table 3. Significant differences from the negative control group were found with the 50 and 100 mg/mL concentrations of the SPDB LCF, but no differences were found with the 25 mg/mL concentration. The three concentrations assessed with the CzDoxB LCF differed with respect to the negative control (*p* < 0.05). The recorded mortalities were higher in the CzDoxB LCF than in the SPDB LCF, reaching close to 97% for the highest concentration evaluated.

In addition, the LC_50_ and LC_90_ values for both media (SPDB and CzDoxB) were estimated. The LC_50_ and LC_90_ values found for CzDoxB were 29.559 and 61.948 mg/mL, respectively, while for SPDB, they were higher (92.687 and 151.363 mg/mL, respectively).

**Figure 5:**
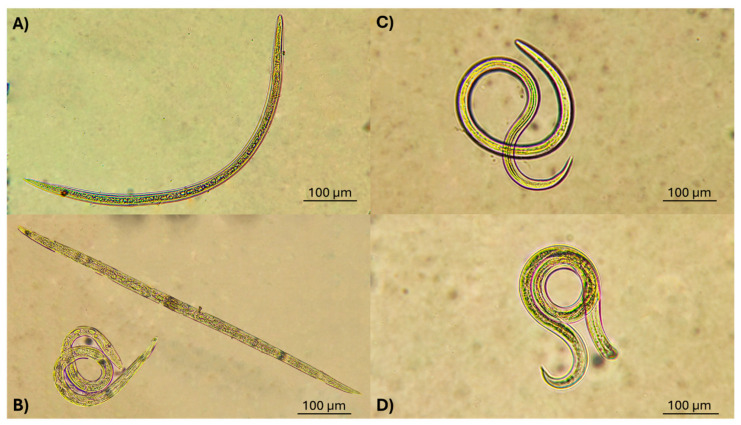
Larvae observed after confrontation with liquid culture filtrates from *Lecanicillium psalliotae* cultured in sweet potato dextrose broth (**A**) and in Czapek Dox broth (**B**), and larvae obtained from the control groups (without fungus culture) (**C**,**D**).

Additionally, microphotographs of larvae recovered from confrontation with *L*. *psalliotae* LCFs and larvae from the control groups (without fungus culture) are shown in Figure 5. Most larvae appeared motionless and lightly curved or stretched, and, after applying a physical stimulus by touching their cuticles, they remained motionless. This confirmed that these larvae were dead. No other morphological changes, either internal or on the surface coat, were observed. In contrast, non-exposed larvae showed vigorous, rapid, and active swimming movements on the medium. These findings were evidenced through the photographs shown in Figure 5A–D.

### 3.5. Myco-Chemical Profile

The results obtained from the chemical group profiles by reagents are shown in Table 4. Alkaloids and tannins were the groups of compounds identified in this study, and these compounds were found in both LCFs, SPDB and CzDoxB.

## 4. Discussion

Macroscopic observation showed that the fungal cultures on PDA plates turned white and cottony. Additionally, the reverse side of the agar plates changed to a light reddish colour. This result gave us an indication of the possible genus/species of the fungus. Likewise, under microscopic observation, we identified a verticillate fungus. Looking at the available literature about verticillate fungi, in 2001, Zare and Gams [33] described a number of species of the genus *Verticillium* that were moved to the *Lecanicillium* genus, and this list included the species *L*. *psalliotae*. These authors mentioned that this species is able to turn the colour of the agar red. These authors made a brief microscopic description of this species and found that it turned the agar from red to purple after 10 days of growing in PDA, usually diffusing into the agar. Additionally, they reported the presence of phialides that were rather long and aculeate and arose from undifferentiated prostrate conidiophores, as well as the presence of macro- and microconidia. They reported the presence of macroconidia that were typically solitary and usually falcate; subsequently, oval to ellipsoidal microconidia were observed [33]. When we observed our isolate in detail, we found that these characteristics matched those of our fungus. Even the conidia and phialide dimensions they reported were similar to those found in our isolate. The analysis of the information previously described for this species led us to morphologically classify it as *L*. *psalliotae*. Species of verticillate fungi are very similar, and they can be easily confused morphologically. Molecular taxonomy is a very useful tool that can be used to confirm a diagnosis. There are some discussions and discrepancies among taxonomists regarding the differentiation between some verticillated fungi, such as *L*. *saksanae* and *L*. *psalliotae*. In the phylogenetic tree that we built, the sequence of our isolate appears in the same clade as *L*. *psalliotae* (KF358375) and *L*. *saksenae* (MH861374), and these relationships were highly supported (BT = 95 and 76%; PP = 1), confirming the morphological findings. When *L*. *saksenae* was described for the first time, the authors mentioned the production of red pigment as the only morphological difference of *L*. *psalliotae* in comparison with *L*. *saksanae* [33,38]. In contrast, Sreeja et al. (2023) [39] reported a strain from *Lecanicillium saksenae* that produced a wine-red pigment, oosporein, which is an insecticidal metabolite produced by *L*. *psalliotae* and other entomopathogenic fungi [40,41,42]. In this regard, we think that the characteristic of turning the colour of the culture medium in both species, as well as their very similar morphological characteristics, could suggest that they are the same species but in a transitional stage between both. However, this is only a hypothesis that will need to be proved in future studies. The results of this assay showed evidence that *L. psalliotae* released bioactive compounds in both liquid culture media, and H. contortus eggs were highly susceptible to the effect of this material, particularly when eggs were exposed to the highest concentrations. In the case of LCF obtained from SPDB, concentrations higher than 15 mg/mL resulted in an ovicidal effect of >83%; meanwhile, in CzDoxB, concentrations higher than 20 mg/mL resulted in ovicidal activity of >87%. The highest ovicidal activity of the LCFs was obtained in both cases at the highest concentrations (25 mg/mL; 97.2% for SPDB and 99.06% for CzDoxB). In an extensive review of the available literature, the authors found only a few studies assessing LCFs or extracts obtained from other genera/species of nematophagous fungi against eggs of trichostrongylids, and no details about morphological changes due to the exposure of eggs to these materials were described [43]. In an experiment carried out in Brazil using LCFs obtained from three nematode-antagonist fungi, *Paecilomyces lilacinum*, *Trichoderma harzianum,* and *T*. *virens*, cultured in a defined medium containing NH_4_NO_3_, MgSO_4_ 7H_2_O, NaHPO_4_ 7H_2_O, KH_2_PO_4_, yeast extract, and gelatine, the following egg-hatching inhibition mortalities were found after 24 h of exposure: *Ancylostoma* sp., *P*. *lilacinum* 44.73%; *T*. *harzianum* 45.29%; and *T*. *virens* 43.57% [43]. In another study on LCFs obtained from *Pochonia chlamydosporia* cultured in a defined medium containing NaCl, MgSO_4_ 7H_2_O, K_2_HPO_4_, yeast extract, and gelatine, the authors reported 72.8% egg-hatching inhibition against cyathostomins (a horse parasitic group) after 24 h of exposure [44]. Studies on agriculturally important nematodes have reported the ovicidal activity of nematophagous fungi with different results. In a study performed in Pakistan, Mukhtar and Pervaz (2003) [45] assessed filtrates from *P*. *chlamydosporia* grown in potato dextrose broth against *M*. *incognita* eggs and reported 84% egg-hatching inhibition after 48 h of exposure. In an experiment assessing the nematocidal activity of the verticillate fungus *Verticillium leptobactrum* against the root-knot nematode *M*. *incognita*, the results showed an egg-hatching inhibition of 50% compared with a control group, and the eggs showed extensive damage and shell degradation with pronounced shell peeling. The authors reported that all treated eggs showed shrinkage, suggesting structural collapse with a loss of turgor [46]. However, at this time, the mode of action and the causes of the morphological changes observed in the *H*. *contortus* eggs attributed to the effect of *L*. *psalliotae* LCF cannot be explained. Future studies using environmental scanning electron microscopy (ESEM) and confocal laser microscopy (CLSM) of eggs exposed to LCF could be crucial in identifying the presence of compounds affecting the egg structures of this important parasite and may explain their mode of action [47]. The *L*. *psalliotae* LCFs cultured in both liquid media showed high egg-hatching inhibition percentages for *H*. *contortus*, particularly at the highest concentration assessed (25 mg/mL). We found this important activity using only LCF that contained a large number of compounds derived from the secondary metabolism of *L*. *psalliotae*. In this context, it is important to elucidate the compound(s) responsible for the nematocidal activity. Our research group is planning to investigate this by separating and fractioning compounds using chromatographic tools, such as high-performance liquid chromatography (HPLC), gas chromatography/mass spectrometry (GC/MS), and nuclear magnetic resonance (NMR) [48]. There are a limited number of studies assessing the nematocidal activity of the LCFs of nematophagous fungi. A few studies have looked at the effects of organic extracts obtained from groups of nematophagous fungi belonging to Orbiliales and Cordycipitaceae on infective larvae of *H*. *contortus*, with encouraging results. For example, in one experiment, fungal extracts obtained from four fungi cultured in potato dextrose broth with methanol/dichloromethane (70/30) resulted in important larval mortalities as follows: *Arthrobotrys oligospora* 72%, *A*. *conoides* 81.6%, *A*. *arthrobotryoides* 89.5%, and *Purpureocillium lilacinum* 78% [49]. In another study, spores of the nematode-trapping fungus *A*. *musiformis* in an aqueous suspension were orally administered to sheep naturally infected with gastrointestinal parasitic nematodes, and a 57.4% faecal egg reduction was recorded. These results suggest that compounds present in LCF exerted ovicidal activity [50]. The authors of the present study could not find additional publications regarding the nematocidal activity of LCFs of nematophagous fungi against *H*. *contortus* or other parasites belonging to the Trichostrongylidae group.

In an extensive review of the available literature, the authors of the current study did not find any record of the presence of alkaloids and tannins in L. psalliotae; therefore, this study could represent the first such evidence. The two groups of compounds were identified in the LCFs of the fungus cultured in both media. Alkaloids have been found in other verticillate fungi, i.e., Pochonia chlamydosporia [51,52]. This species is considered an important ovicidal nematophagous fungus and an effective biotechnological tool against phytoparasitic nematodes well known in the agricultural industry [53]. The production of a large list of compounds, including tannins, flavonols, and saponins, was found in aqueous and ethanolic extracts of Duddingtonia flagrans [54], and this species has been widely studied as a potential biotechnological tool for ruminant parasitic nematodes [55]. Likewise, tannins produced by a large list of plants have been reported as potential biological control tools against gastrointestinal parasitic nematodes in ruminants [56]. Interestingly, the alkaloids and tannins found in the present study have been found in a group of plants and have shown important nematocidal activity against animal parasitic nematodes, particularly in sheep and goats [57,58]. These reports suggest that both groups of myco-compounds we identified in L. psalliotae could be responsible for the nematocidal activity in the present study. These groups of compounds are important to consider in future studies focused on finding a practical use of natural compounds with nematocidal activity. Nevertheless, identifying the specific compound(s) responsible for the nematocidal activity is important, and this can be achieved using chromatographic techniques, i.e., HPLC, GC/MS, and NMR.

## 5. Conclusions

The results of this study lead us to conclude that the nematophagous fungus *L*. *psalliotae* produces nematocidal compounds against the eggs and larvae of the blood-feeding nematode *H*. *contortus*. The myco-chemical analysis of groups of compounds indicated the presence of alkaloids and tannins. These compounds were released by *L*. *psalliotae* into two liquid culture media (SPDB and CzDoxB), and the liquid culture filtrates exerted important in vitro nematocidal activity against *H*. *contortus*, one of the most pathogenic agents affecting the livestock industry.

## Figures and Tables

**Table 1:** Measurements of the main morphological characteristics of taxonomic importance recorded for identification of the *Lecanicillium psalliotae* INIFAP-STp-01 strain.

Characteristic	Mean	Range
Phialide length (µm)	21.75	15.6–33.8
Phialide width (µm)	1.78	1.26–2.52
Macroconidia length (µm)	9.06	6.74–11.94
Macroconidia width (µm)	2.08	1.56–2.61
Microconidia length (µm)	4.94	4.27–5.73
Microconidia width (µm)	1.67	1.25–2.04
Phialides/whorl	3	1–5

**Table 2:** Means and standard errors of recovered eggs/pre-infective larvae of *Haemonchus contortus* and the egg hatching inhibition percentage after 48 h of confrontation with different concentrations of fungal liquid culture filtrates from *Lecanicillium psalliotae* in sweet potato dextrose broth (SPDB) and Czapek Dox Broth (CzDoxB).

Media	Concentration (mg/mL)	Larvae (Mean)	Eggs (Mean)	Total Count (Mean)	Egg-Hatching Inhibition (Mean ± SE) *
SPDB	0	127.56	2.45	130.25	1.88 ± 1.14 ^a^
	10	53.34	71.23	125.00	56.98 ± 15.40 ^b^
	15	19.78	99.55	119.00	83.66 ± 6.03 ^bc^
	20	6.13	128.68	134.75	95.49 ± 1.80 ^c^
	25	2.67	99.51	102.38	97.20 ± 0.80 ^c^
CzDoxB	0	115.32	1.63	117.00	1.39 ± 0.60 ^a^
	10	36.09	54.70	90.75	60.28 ± 4.00 ^b^
	15	33.54	67.86	101.38	66.94 ± 4.45 ^b^
	20	13.76	94.58	108.57	87.11 ± 3.05 ^c^
	25	0.97	103.27	104.25	99.06 ± 0.20 ^c^

NF = No Fungus culture; * Different letter shows statistical differences between concentrations (*p* < 0.05).

**Table 3:** Means and standard errors of live and dead *Haemonchus contortus* infective larvae recovered after 48 h confrontation with liquid culture filtrates from *Lecanicillium psalliotae* grown in sweet potato dextrose broth (SPDB) and Czapek Dox Broth (CzDoxB) at different concentrations.

Media	Concentration (mg/mL)	Dead/Total Larvae (Mean)	Larval Mortality (%) (Mean ± SE) *
SPDB	NF	4.42/90.67	4.87 ± 1.05 ^a^
	25	3.98/101.09	3.94 ± 0.77 ^a^
	50	19.96/96.63	20.66 ± 1.53 ^b^
	100	58.77/108.29	54.27 ± 4.56 ^c^
CzDoxB	NF	3.32/83.38	3.98 ± 0.74 ^a^
	25	44.23/94.45	46.82 ± 7.15 ^b^
	50	69.77/76.50	91.20 ± 2.80 ^c^
	100	85.27/88.09	96.80 ± 0.68 ^c^

NF = No fungus; SE = Standard error; * Different letters in the same medium show significant differences between concentrations (*p* < 0.05). n = 4.

**Table 4:** Myco-chemical groups present in the liquid culture filtrates from *Lecanicillium psalliotae*.

Metabolite and Reagent	Colorimetric Reaction	*Lecanicillium psalliotae*	
		SPDB	CzDoxB
Alkaloids Dragendorff Mayer Wagner	Turbidity or precipitate (red to orange, white to cream, and brown)	+++ + ++	+++ + +
Coumarins Bornträger	Yellow fluorescence (U.V)	-	-
Flavonoids Mg^2+^ and HCL	Red, orange, and violet colour	-	-
Tannins Ferric chloride (FeCl_3_)	Hydrolysable tannins (blue) Condensed tannins (green)	+++ +	+ ++
Confirmation			
Solution of gelatine	Precipitate white	+	+
Gelatine and saline solution	Precipitate white	+	+
Saline solution	Precipitate white	-	-
Triterpenes/Sterols Liebermann–Buchard	Colour blue, blue-green (sterols)	-	-
Salkowski	Red to purple (triterpene)	-	-
Saponins Water	Foam formation	-	-

(-) Not detected; (+) light positive reaction; (++) positive reaction; (+++) strong positive reaction. SPDB = sweet potato dextrose broth; CzDoxB = Czapek Dox Broth.

## Data Availability

Data are contained within the article.
